# Dynamics in bioprocess development for *Pichia pastoris*

**DOI:** 10.4161/bioe.36152

**Published:** 2014-10-30

**Authors:** Oliver Spadiut, Christoph Herwig

**Affiliations:** Vienna University of Technology; Institute of Chemical Engineering; Research Area Biochemical Engineering; Vienna, Austria

**Keywords:** *Pichia pastoris*, dynamics, bioprocess development, fed-batch, physiological parameter, specific substrate uptake rate

## Abstract

P*ichia pastoris* is a widely used host organism for the recombinant production of proteins. It is attracting increasing interest for the production of biopharmaceuticals, due to its capability of performing posttranslational modifications. Traditionally, production processes with *P. pastoris* describe fed-batch processes based on feed forward regimes with a constant specific growth rate. However, this strategy does not consider physiological changes of the organism, bearing the risk of overfeeding and thus harming the cells. Recently, we introduced the specific substrate uptake rate as a novel physiological parameter to design fed-batch strategies for *P. pastoris*. We showed that by doing dynamic batch experiments strain specific parameters, which are needed to set up respective feeding profiles, can be easily determined. Furthermore we proved that dynamics during feeding directly affects productivity and product purity. Here, we sum up our findings regarding dynamics in bioprocess development for *P. pastoris*.

## Abbreviations

μ: specific growth rate
q_s_: specific substrate uptake rate
q_p_: specific productivity
C-source: carbon-source

## Introduction

The methylotrophic yeast *Picha pastoris* is a very useful microbial host for the production of complex proteins and biopharmaceuticals due to its capability of performing post-translational modifications. Traditionally, fed-batch production processes with this yeast are based on the specific growth rate (μ) and are mostly done by applying feed forward regimes with constant μ resulting in exponential feed profiles.^1,2^ This strategy describes a rather rigid system, where cells are always exposed to basically the same conditions. However, this strategy does not consider any physiological changes of the culture, like changes in yields or the maximum substrate uptake capacity, bearing the risk of overfeeding and thus harming the cells. In previous studies, we introduced the specific substrate uptake rate (q_s_) as a novel physiological key parameter to design fed-batch strategies for *P. pastoris*. We showed that by doing dynamic experiments in batch cultivation important physiological information on the respective *P. pastoris* strain can be gathered in a fast and simple manner.^3-6^ Furthermore we showed that applying dynamics during fed-batch does not only give more detailed information on cell physiology and capacity but also affects productivity and product purity. Here we summarize our findings, show the different opportunities of applying dynamics in bioprocess development with *P. pastoris* and underline the great potential in doing so.

## Dynamics in Batch Cultivation

As we repeatedly showed in previous studies, useful physiological information on *P. pastoris* can be gathered in a rather simple and fast manner by applying substrate pulses during dynamic batch cultivation. Sampling before and after each pulse and subsequent sample analysis allows the calculation of strain specific parameters. This strategy can be applied for different purposes.

### Physiology during adaptation to a new substrate

**Figure 1.**
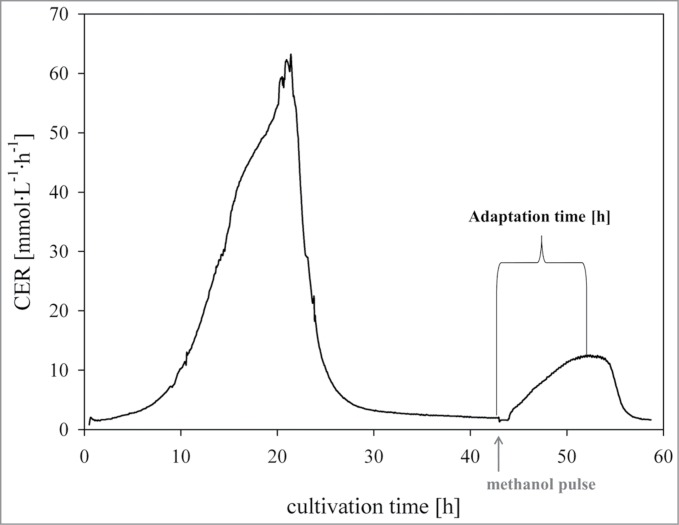
Batch phase and methanol adaptation pulse. Sampling before and after the pulse allows the calculation of the substrate uptake rate during the adaptation phase (q_s adapt_). Black line, carbon dioxide evolution rate.

After a batch phase on the C-source of choice, like glucose or glycerol, another C-source, like methanol, is pulsed. The time from applying the pulse until the maximum in the offgas signal is reached can be regarded as the adaptation time of the cells to the new substrate (**Fig. 1**). The adaptation time is especially relevant, if a subsequent fed-batch is envisioned and the second substrate is potentially harmful for the cells. Without knowing the adaptation time of the *P. pastoris* strain to the second substrate, one runs the risk of overfeeding the cells in the fed-batch cultivation. Previously, we showed that different *P. pastoris* strains showed very different characteristics during adaptation to methanol underlining the great need of individual strain analysis during this phase.^7^

Furthermore, the uptake rate for the second substrate during the adaptation phase (q_s adapt_) can be easily determined by this method.^4,7^ This value should not be exceeded during the adaptation time in the subsequent fed-batch to avoid substrate accumulation.

The importance of the adaptation phase of *P. pastoris* to methanol, which still describes the main inducer for this methylotrophic yeast, was also investigated in detail by Jungo et al.^8^ The authors analyzed the activity of the intracellular enzyme alcohol oxidase and correlated this to the uptake of methanol and toxic methanol accumulation. These experiments were done in time-consuming continuous cultivations, whereas it is possible to obtain the same information by doing dynamic batch experiments. By pulsing 0.5% (v/v) methanol and sampling before and after the pulse as well as monitoring the offgas signal, q_MeOH adapt_ and the adaptation time can be determined^4^ (**Fig. 1**).

### Determination of the maximum specific substrate uptake rate (q_s max_)

By applying at least three consecutive substrate pulses at a final concentration below a potentially inhibiting or even toxic level, the maximum uptake rate of the cells for this substrate can be determined. This value is crucial for the subsequent fed-batch design since it describes the maximum feeding set point.^7^

### Identification of useful C-sources

**Figure 2.**
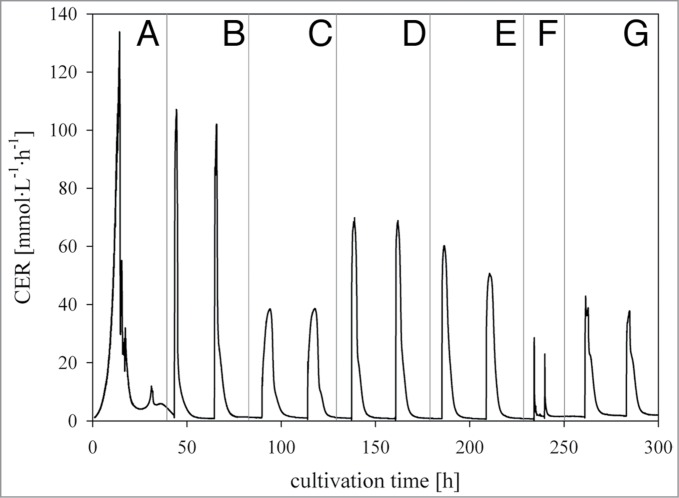
Batch cultivation with repeated pulses of different C-sources. (**A**) Batch on glucose; followed by two consecutive pulses of (**B**) glucose; (**C**) sorbitol; (**D**) mannose; (**E**) fructose; (**F**) maltose; (**G**) glycerol, at a final concentration of 45 mM. Black line, carbon dioxide evolution rate.

The strategy of applying substrate pulses during batch cultivation is also useful to identify potentially useful C-sources for *P. pastoris* and the respective physiological answer of the yeast to the different substrates as adaptation time, uptake rates and metabolite formation can be easily investigated. In **Figure 2** the carbon dioxide evolution rate of a *P. pastoris* strain cultivated in batch, where different C-sources at a final concentration of 45 mM were consecutively pulsed, is shown. Sampling and sample analysis allow the determination of the specific uptake rate for the respective substrate and the formation of potential metabolites.

### Physiology at different cultivation conditions

The dynamic pulse strategy is especially useful for a fast screening of cultivation conditions. By applying at least two consecutive substrate pulses at the respective conditions, for example pH, temperature, and dissolved oxygen concentration can be varied, specific substrate uptake rate, metabolite formation, and specific productivity (q_p_) can be determined. Thus, the cells can be physiologically characterized at different cultivation conditions in a fast and easy way.

## Dynamics in Fed-Batch Cultivation

In contrast to the rigid feed forward fed-batch system based on constant μ, we showed that dynamic feeding affects both productivity and product purity and can furthermore be very useful to obtain more detailed information on strain physiology and capacity.^4,7,9,10^

### Effect on productivity

We repeatedly showed that dynamic stepwise feeding from q_s adapt_ to a value close to q_s max_, which were both determined in dynamic batch experiments before, give higher specific and volumetric productivities compared with a feeding strategy based on constant μ.^4,9^ For some *P. pastoris* strains it was possible to increase the specific productivity at least 2-fold using the dynamic feeding regime compared with a more static one. Furthermore we found out that stepwise increasing the feed rate is more beneficial than linear ramps.^9^ Apparently cells secrete more product when they are challenged by a stepwise increase of the feed rate but then again have time to adapt to culture conditions.

### Effect on product purity

In terms of product purity we found out that stepwise feeding regimes, which describe more stress for the cells than linear feeding strategies, not only cause the cells to secrete more product but unfortunately also more contaminating proteins.^9^ Thus, bioprocess engineers have to decide how pronounced the dynamics during the fed-batch should be, as steps in the feed rate cause more productivity but lower product purity, whereas linear ramps from q_s adapt_ to q_s max_ cause lower productivity but higher product purity. A crucial decision-making point in this respect is the available downstream process for the product.

### Cell physiology and capacity

**Figure 3.**
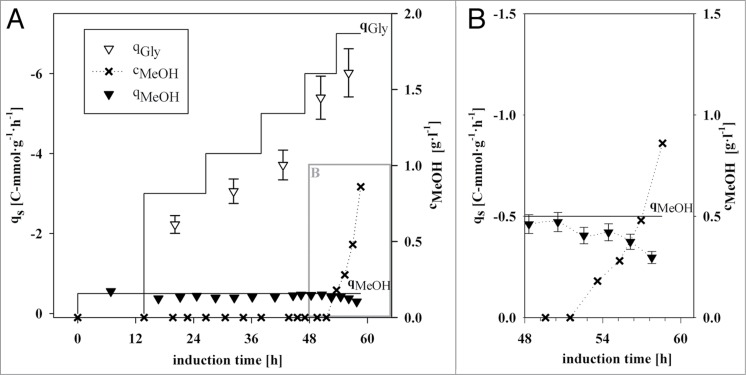
Methanol accumulation at high q_s glycerol_ setpoints. **(A**) Experimental strategy and calculated specific uptake rates. (**B**) Zoom in of the last phase of the experiment—marked with a gray box in figure A—where methanol accumulated. Figure taken with permission from reference 10.

The highest potential for applying dynamics during a fed-batch lies in the development of mixed feed strategies for *P. pastoris*. Mixed feed strategies give certain technical benefits, like lower oxygen consumption and heat production,^11^ and facilitate biomass growth due to a higher biomass yield on the second substrate.^12^ We showed the great potential of dynamics during fed-batch for the development of an efficient and robust glycerol-methanol mixed feed system. The feed rate of methanol was adjusted to correspond to a constant q_s methanol_ allowing full induction, whereas the feed rate of glycerol was dynamically increased in steps of q_s glycerol_.^10^ Concomitantly, methanol accumulation in the cultivation broth was monitored and thus a critical specific glycerol uptake rate, where methanol accumulated, could be determined in only one fed-batch experiment (**Fig. 3**). Furthermore, the specific productivity (q_p_) at the different q_s glycerol_ steps was analyzed and thus another critical q_s glycerol_ level, where a decline of q_p_ occurred, could be determined. Consequently, using this dynamic approach a substrate mixing ratio allowing highest productivity could be identified. Summarizing, the dynamic q_s_-controlled strategy allowed the concomitant characterization of metabolism and recombinant protein production in only one experiment.

In conclusion, we strongly recommend using dynamics in bioprocess development, where the major objective is the identification of process parameters allowing highest concentration and quality of the desired product. Traditionally, numerous fed-batch experiments and time-consuming continuous cultivations have to be conducted for this purpose. On the contrary, using dynamics in either batch or fed-batch cultivations allows:

- fast and simple identification of a minimal set of parameters needed to set up consecutive fed-batch regimes based on the physiological parameter q_s_
- fast and simple screening of different *P. pastoris* strains, cultivation conditions, and C-sources
- increased productivity compared with more rigid feeding regimes
- detailed physiological characterization in mixed feed environments and consequent fast development of mixed substrate feeding strategies
- increased process understanding and optimization of product formation in only a few experiments allowing fast process development

### Disclosure of Potential Conflicts of Interest

No potential conflicts of interest were disclosed.

## References

[1] Potgieter TI, Cukan M, Drummond JE, Houston-Cummings NR, Jiang Y, Li F, Lynaugh H, Mallem M, McKelvey TW, Mitchell T, et al. Production of monoclonal antibodies by glycoengineered Pichia pastoris. J Biotechnol 2009; 139:318-25; PMID:19162096; http://dx.doi.org/10.1016/j.jbiotec.2008.12.015 19162096

[2] Trinh LB, Phue JN, Shiloach J. Effect of methanol feeding strategies on production and yield of recombinant mouse endostatin from Pichia pastoris. Biotechnol Bioeng 2003; 82:438-44; PMID:12632400; http://dx.doi.org/10.1002/bit.10587 12632400

[3] Spadiut O, Dietzsch C, Herwig C. Determination of a dynamic feeding strategy for recombinant Pichia pastoris strains. Methods Mol Biol 2014; 1152:185-94; PMID:24744034; http://dx.doi.org/10.1007/978-1-4939-0563-8_11 24744034 PMC4826592

[4] Dietzsch C, Spadiut O, Herwig C. A dynamic method based on the specific substrate uptake rate to set up a feeding strategy for Pichia pastoris. Microb Cell Fact 2011; 10:14; PMID:21371310; http://dx.doi.org/10.1186/1475-2859-10-14 21371310 PMC3059269

[5] Krainer FW, Dietzsch C, Hajek T, Herwig C, Spadiut O, Glieder A. Recombinant protein expression in Pichia pastoris strains with an engineered methanol utilization pathway. Microb Cell Fact 2012; 11:22; PMID:22330134; http://dx.doi.org/10.1186/1475-2859-11-22 22330134 PMC3295664

[6] Krainer FW, Gmeiner C, Neutsch L, Windwarder M, Pletzenauer R, Herwig C, Altmann F, Glieder A, Spadiut O. Knockout of an endogenous mannosyltransferase increases the homogeneity of glycoproteins produced in Pichia pastoris. Sci Rep 2013; 3:3279; PMID:24252857; http://dx.doi.org/10.1038/srep03279 24252857 PMC3834888

[7] Dietzsch C, Spadiut O, Herwig C. A fast approach to determine a fed batch feeding profile for recombinant Pichia pastoris strains. Microb Cell Fact 2011; 10:85; PMID:22032177; http://dx.doi.org/10.1186/1475-2859-10-85 22032177 PMC3214193

[8] Jungo C, Marison I, von Stockar U. Regulation of alcohol oxidase of a recombinant Pichia pastoris Mut+ strain in transient continuous cultures. J Biotechnol 2007; 130:236-46; PMID:17566583; http://dx.doi.org/10.1016/j.jbiotec.2007.04.004 17566583

[9] Spadiut O, Zalai D, Dietzsch C, Herwig C. Quantitative comparison of dynamic physiological feeding profiles for recombinant protein production with Pichia pastoris. Bioprocess Biosyst Eng 2014; 37:1163-72 PMID:24213806; http://dx.doi.org/10.1007/s00449-013-1087-z 24213806 PMC4015061

[10] Zalai D, Dietzsch C, Herwig C, Spadiut O. A dynamic fed batch strategy for a Pichia pastoris mixed feed system to increase process understanding. Biotechnol Prog 2012; 28:878-86; http://dx.doi.org/10.1002/btpr.1551; PMID:22505140 22505140

[11] Jungo C, Marison I, von Stockar U. Mixed feeds of glycerol and methanol can improve the performance of Pichia pastoris cultures: A quantitative study based on concentration gradients in transient continuous cultures. J Biotechnol 2007; 128:824-37; PMID:17303281; http://dx.doi.org/10.1016/j.jbiotec.2006.12.024 17303281

[12] Jungo C, Rerat C, Marison IW, von Stockar U Quantitative characterization of the regulation of the synthesis of alcohol oxidase and of the expression of recombinant avidin in a Pichia pastoris Mut(+) strain. Enzyme Microb Technol 2006; 39:936-44; http://dx.doi.org/10.1016/j.enzmictec.2006.01.027

